# Phase behavior of silica-PNIPAm nanogels under high hydrostatic pressure

**DOI:** 10.1107/S1600576725003188

**Published:** 2025-05-12

**Authors:** Nele N. Striker, Christina Krywka, Claudia Goy, Svenja C. Hövelmann, Niels C. Giesselmann, Florian Schulz, Irina Lokteva, Fabian Westermeier, Frederic Caupin, Michael Paulus, Felix Lehmkühler

**Affiliations:** a Deutsches Elektronen-Synchrotron DESY Notkestr. 85 22607Hamburg Germany; b Helmholtz-Zentrum Hereon, Institute for Materials Physics, Max-Planck-Str. 1, 21502Geesthacht, Germany; c Institute of Experimental and Applied Physics, Kiel University, Leibnizstraße 19, 24118Kiel, Germany; d Fachbereich Physik, Universität Hamburg, Luruper Chaussee 149, 22761Hamburg, Germany; e The Hamburg Centre for Ultrafast Imaging, Luruper Chaussee 149, 22761Hamburg, Germany; f Institut Lumière Matière Université Claude Bernard Lyon 1, CNRS, Institut Universitaire de France F-69622Villeurbanne France; g Fakultät Physik/DELTA, TU Dortmund, 44221Dortmund, Germany; Argonne National Laboratory, USA

**Keywords:** X-ray photon correlation spectroscopy, XPCS, PNIPAm nanogels, high pressure, small-angle X-ray scattering, SAXS, volume phase transitions

## Abstract

The structure and dynamics of concentrated silica-PNIPAm (poly-*N*-iso­prop­yl­acrylamide) nanogels are studied as a function of hydrostatic pressure up to 3500 bar. A combination of X-ray photon correlation spectroscopy and small-angle X-ray scattering shows similar characteristics of the dynamics for both temperature- and pressure-induced volume phase transitions of PNIPAm nanogels. Upon increasing the pressure and depending on the initial particle volume fraction, a transition from a liquid or glass state to a colloidal gel is observed for pressures *p* ≃ 1500 bar at a temperature of 293 K. Time-dependent analysis of the dynamics shows aging in glass and gel samples which is absent in the liquid state. This indicates stress-dominated dynamics upon pressure changes that equilibrate after a few hundred seconds.

## Abstract 2

The effect of hydrostatic pressure on the structure and dynamics of concentrated silica-PNIPAm (poly-*N*-isopropylacrylamide) nanogels reveals characteristics similar to those found in temperature-induced phase transitions. However, significant aging is seen in glass and gel samples, which is absent in the liquid state.

## 1. Introduction

Stimuli-responsive polymers are materials that undergo a volume phase transition, typically via a coil-to-globule transition, as a response to changes in their environment such as temperature, pressure or pH. Among these materials, poly-*N*-isopropylacrylamide (PNIPAm) is the most popular and most frequently studied system with many potential applications (Stuart *et al.*, 2010 ▸; Koetting *et al.*, 2015 ▸). In particular, PNIPAm is well known for its lower critical solution temperature (LCST) of around 305 K which promises applications in biology and medicine (Halperin *et al.*, 2015 ▸; Lanzalaco & Armelin, 2017 ▸; Shaibie *et al.*, 2023 ▸) as well as sensing and actuation (Hu *et al.*, 2021 ▸; Liu *et al.*, 2022 ▸). Aside from the studies of PNIPAm hydrogels, micro- or nanogels of PNIPAm are the focus of research (Das *et al.*, 2006 ▸; Yunker *et al.*, 2014 ▸; Karg *et al.*, 2019 ▸; Brijitta & Schurtenberger, 2019 ▸). When crossing the LCST, PNIPAm becomes insoluble in water. As a consequence, the microgel particles expel water and thus shrink in size. From a fundamental point of view, such microgel particles have been used to investigate the phase diagram of soft particles in general (Mattsson *et al.*, 2009 ▸; Philippe *et al.*, 2018 ▸; Frenzel *et al.*, 2021 ▸). Below the LCST the interaction in the colloidal system is described best by repulsive Hertzian potentials (Bergman *et al.*, 2018 ▸), resulting in liquid, glassy or crystalline phases (Paloli *et al.*, 2013 ▸; Philippe *et al.*, 2018 ▸; Frenzel *et al.*, 2021 ▸). In contrast, above the LCST the particles become attractive, forming colloidal gels for a broad range of volume fractions (Zaccone *et al.*, 2011 ▸; Zaccone *et al.*, 2013 ▸; Frenzel *et al.*, 2019 ▸; Frenzel *et al.*, 2021 ▸). Furthermore, in the swollen state below the LCST they also allow access to overpacked concentrations with high degrees of softness (Scotti *et al.*, 2019 ▸; Scotti *et al.*, 2022 ▸).

Apart from temperature, PNIPAm shows a response to pressure (Lee *et al.*, 1990 ▸; Otake *et al.*, 1993 ▸; Shibayama *et al.*, 2004 ▸; Papadakis *et al.*, 2023 ▸). With increasing pressure, the LCST first shifts to higher temperatures with a maximum at 600 bar, and it then decreases for higher pressures (Papadakis *et al.*, 2023 ▸). At *T* = 293 K a pressure of approximately 2000 bar is needed to induce the volume phase transition (Niebuur *et al.*, 2020 ▸). So far, experimental studies have focused on structural properties due to pressure-induced coil-to-globule transitions, *e.g.* using small-angle neutron scattering (SANS) (Shibayama *et al.*, 2004 ▸; Niebuur *et al.*, 2018 ▸; Niebuur *et al.*, 2020 ▸) or small-angle X-ray scattering (SAXS) (Grobelny *et al.*, 2013 ▸), and properties of hydration water by quasi-elastic neutron scattering (Osaka *et al.*, 2009 ▸; Niebuur *et al.*, 2019*b* ▸). More details can be found in the review by Papadakis *et al.* (2023 ▸).

While many studies show that similar volume transitions appear as a response to pressure or temperature increase (Kunugi *et al.*, 2005 ▸), the underlying mechanisms are fundamentally different (Meersman *et al.*, 2005 ▸). In particular, temperature and pressure increase should result in antagonistic effects with respect to the hydration of PNIPAm. Recent molecular dynamics simulations could demonstrate that the gain of hydration water upon increasing pressure results in an increase of the LCST for low pressures, as discussed above, whereas further increasing the pressure leads to a reduction of chain size as well as a change of the hydration mechanism and thus a decrease of the LCST (Tavagnacco *et al.*, 2021 ▸). However, there is still a lack of theoretical and experimental work to understand the detailed structure, dynamics and the driving forces of the volume phase transition with increasing pressure.

Furthermore, the majority of the research is dedicated to single-particle properties. To the best of our knowledge, there is no study investigating the structure and dynamics of a PNIPAm microgel dispersion at high concentration and different pressures. Here, we address this topic using X-ray photon correlation spectroscopy (XPCS) and SAXS measurements on silica-PNIPAm core–shell particles in dense fluid and glass states, over a pressure range up to 3500 bar. Thus, we use a similar approach as in previous work (Frenzel *et al.*, 2019 ▸; Frenzel *et al.*, 2020 ▸; Nigro *et al.*, 2020 ▸; Frenzel *et al.*, 2021 ▸) where the dynamics and structure of such systems have been tracked as a function of temperature and particle concentration only. Depending on the particle concentration, these studies reported fluid and glass states for the swollen particles at low temperatures and attractive fluid and gel phases above the LCST.

In this work, we study the structure and dynamics of concentrated silica-PNIPAm nanogels at 293 K as a function of hydrostatic pressure. We find that, similar to the effect of temperature, the application of pressure can induce transitions from liquid and glass states to a colloidal gel at around 1500 bar. The glass and gel samples are subject to aging after pressure changes, suggesting stress-dominated dynamics immediately after pressure changes. Our results highlight the need for further studies to reveal the role of pressure on otherwise well studied soft matter systems.

## 2. Experimental

### 2.1. Sample details

The samples used in this experiment are core–shell colloidal nanogels, consisting of a silica core and a PNIPAm outer shell, dispersed in water. Their synthesis is explained by Nun *et al.* (2017 ▸). During synthesis a weight concentration of 4% of methylenbisacrylamide (BIS) was added, acting as a crosslinker connecting two PNIPAm chains. The spherical silica core had a radius of *R*_core_ = 55 nm; the total radius of the particles varies with temperature and induced pressure. In this study, we investigated two different concentrations of the same sample. The higher concentration had a mass fraction of 6.5% and is refered to herein as SP1. The other sample was a 1:1 dilution with water of SP1 and is referred to as SP2.

### 2.2. XPCS

In an XPCS experiment the dynamics of a sample can be studied in real time by means of coherent X-rays (Grübel & Zontone, 2004 ▸; Shpyrko, 2014 ▸; Sandy *et al.*, 2018 ▸; Madsen *et al.*, 2020 ▸; Lehmkühler *et al.*, 2021 ▸). The dynamics are obtained by intensity–intensity correlations of the diffraction patterns, also known as speckle patterns in coherent X-ray scattering experiments, given by 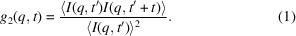
Here, *q* denotes the modulus of the wave vector transfer 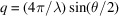, with wavelength λ and scattering angle θ. *I*(*q*, *t*′) is the intensity at a given *q* and time *t*′. Note that the averaging is over detector pixels corresponding to the same *q* (or *q*-bin) and all times *t*′. The *g*_2_-function is related to the intermediate scattering function *f*(*q*, *t*) which contains all information about the time evolution of the sample via the Siegert relation (Ferreira *et al.*, 2020 ▸): 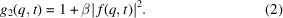
The speckle contrast β is mainly related to the experimental configuration and the coherence properties of the X-ray beam. In many cases, the intermediate scattering function can be described by a Kohlrausch–Williams–Watts (KWW) expression as 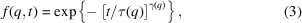
where τ(*q*) is the relaxation time and γ(*q*) is the KWW parameter. The *q* dependence of τ and the value of γ are characteristic of the type of dynamics. For instance, free diffusion is characterized by 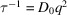 and γ = 1. Here, *D*_0_ is the Stokes–Einstein–Sutherland diffusion coefficient given by 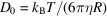 with Boltzmann’s constant *k*_B_, temperature *T*, viscosity η and particle radius *R*.

### 2.3. Experimental setup

**Figure 1:**
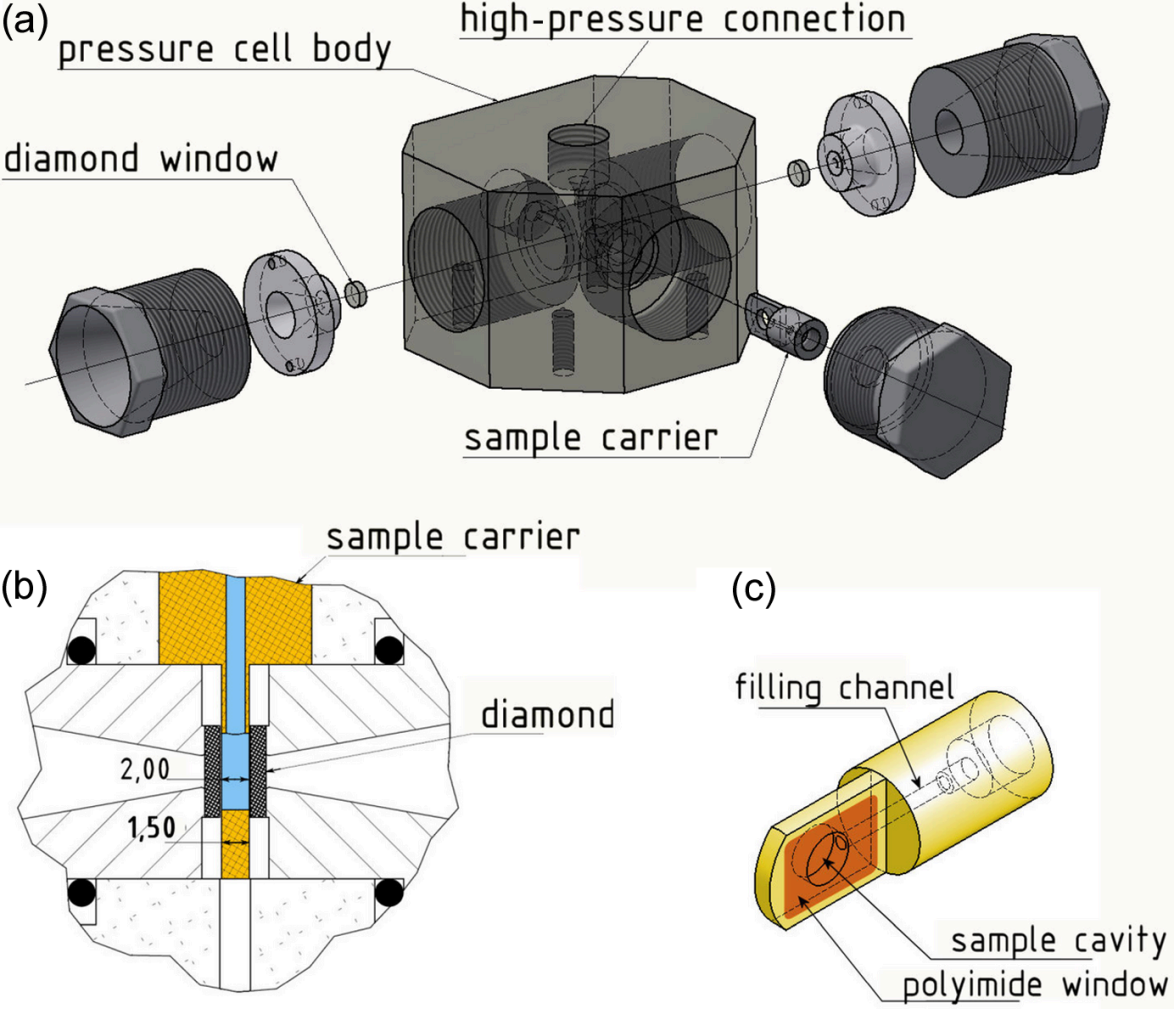
Schematics of the high-pressure cell. (*a*) Side view of the cell. (*b*) Top view of the cell. (*c*) Sample holder.

The XPCS experiment has been performed at beamline P10 of PETRA III at DESY (Hamburg, Germany). An ultra-small angle X-ray scattering (USAXS) geometry was used with a sample–detector distance of 21.2 m. The detector was a Dectris EIGER 500k with a pixel size of 75 µm × 75 µm and a maximum frame rate of 9 kHz. The beam size was set to 100 µm × 100 µm at the sample position, defined by slits. The samples were put in a high-pressure sample environment that allows experiments on soft matter materials up to 7 kbar hydrostatic pressure, similar to the cell presented by Krywka *et al.* (2008 ▸). The schematics of the cell are shown in Fig. 1 ▸. The X-ray beam goes through the length of the chamber and passes two diamond windows, while the sample is mounted from the side. A photon energy of 13 keV was chosen to reduce the X-ray absorption through the 2 mm-thick diamond windows of the pressure cell. The samples were filled into dedicated sample holders with a thickness of 1.5 mm, shown in Fig. 1 ▸(*c*). After the sample holders have been positioned in the high-pressure chamber, XPCS runs were taken at different sample spots at 100 bar with an exposure time of 0.012 s. Afterwards, the pressure was raised in different steps by a hand-driven pump, typically by 500 bar if not labeled differently, and XPCS runs were taken on at least 10 different sample spots to minimize radiation damage. After measuring at 3500 bar, further runs ware taken in a similar fashion during pressure release. Considering the beamline parameters with respect to flux and X-ray transmission of attenuators and sample chamber, the critical dose reported by Lehmkühler *et al.* (2018 ▸) for these systems was achieved after approximately 1 min of measuring time on one spot, limiting the total time of the XPCS series.

## 3. Results

**Figure 2:**
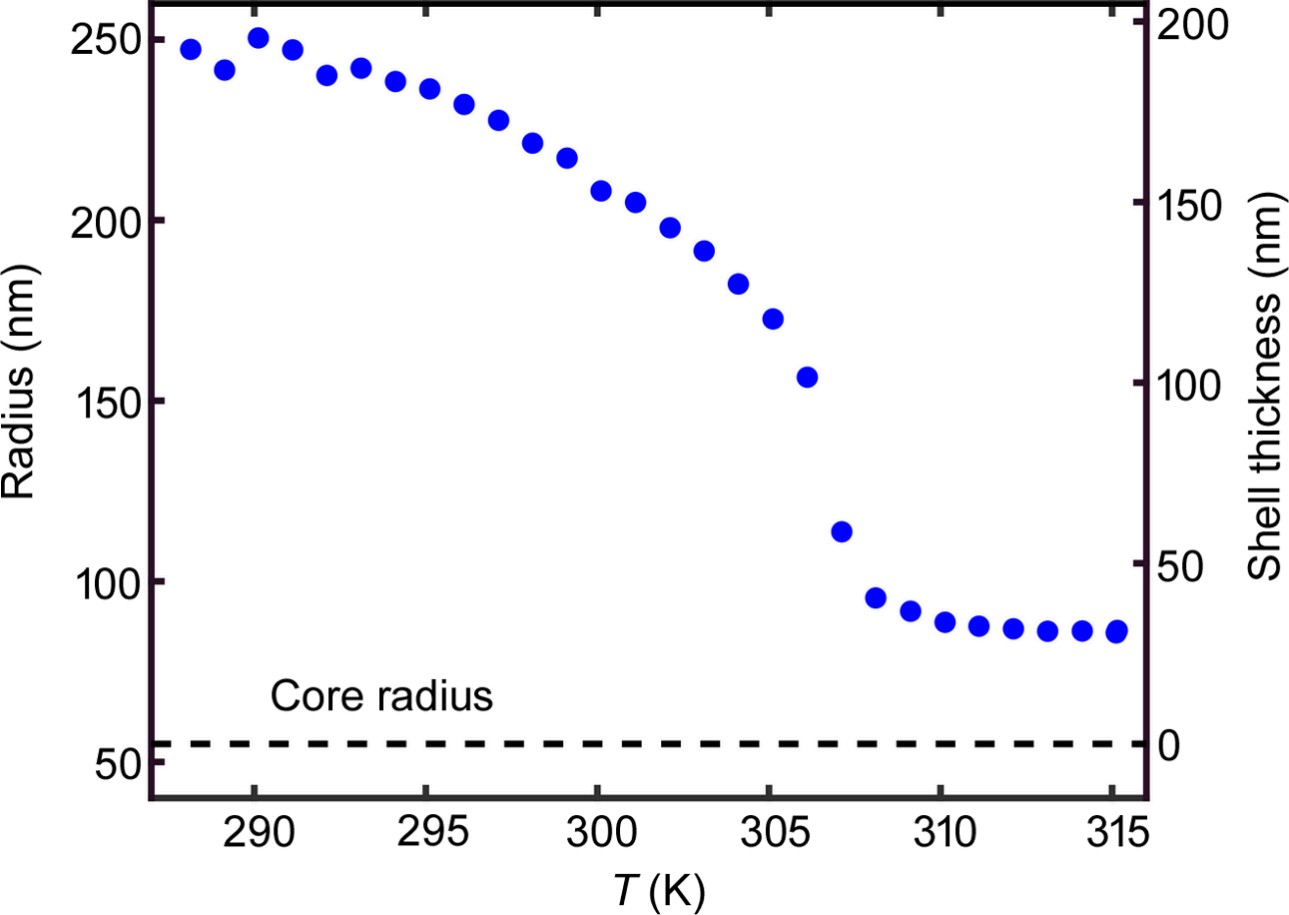
Hydrodynamic radius as a function of temperature of the PNIPAm nanogel. The left axis shows the hydrodynamical radius of the sample, the right axis the corresponding shell thickness. The core radius of the particles is *R*_core_ = 55 nm.

For a first characterization of the sample, it was measured using dynamic light scattering (DLS). The measurements were performed using a 3D-DLS spectrometer–goniometer system from LS Instruments (Fribourg, Switzerland) and a strongly diluted sample. The hydrodynamic radius of the particles at different temperatures (*T*) was determined using the Stokes–Einstein–Sutherland relation and is shown in Fig. 2 ▸. As expected, the sample showed the typical collapse at around 305 K. From these data, the effective volume fractions ϕ of the nanogel system can be calculated. This value is obtained by ϕ = *nV*, with the particle number density *n* and the volume of a single particle in the dilute limit *V* obtained by DLS (Romeo *et al.*, 2010 ▸). Thus, ϕ can also reach values above the random close packing limit and even above 1, with deformed, interpenetrated particle shells or a reduced hydration level (Bouhid de Aguiar *et al.*, 2017 ▸; Mohanty *et al.*, 2017 ▸; Scotti, 2021 ▸). At 315 K the volume fractions are ϕ = 0.048 for SP1 and ϕ = 0.024 for SP2. At 293 K they correspond to ϕ = 1.1 and ϕ = 0.54 for SP1 and SP2, respectively. Thus, SP1 represents an overpacked glassy state at 293 K.

**Figure 3:**
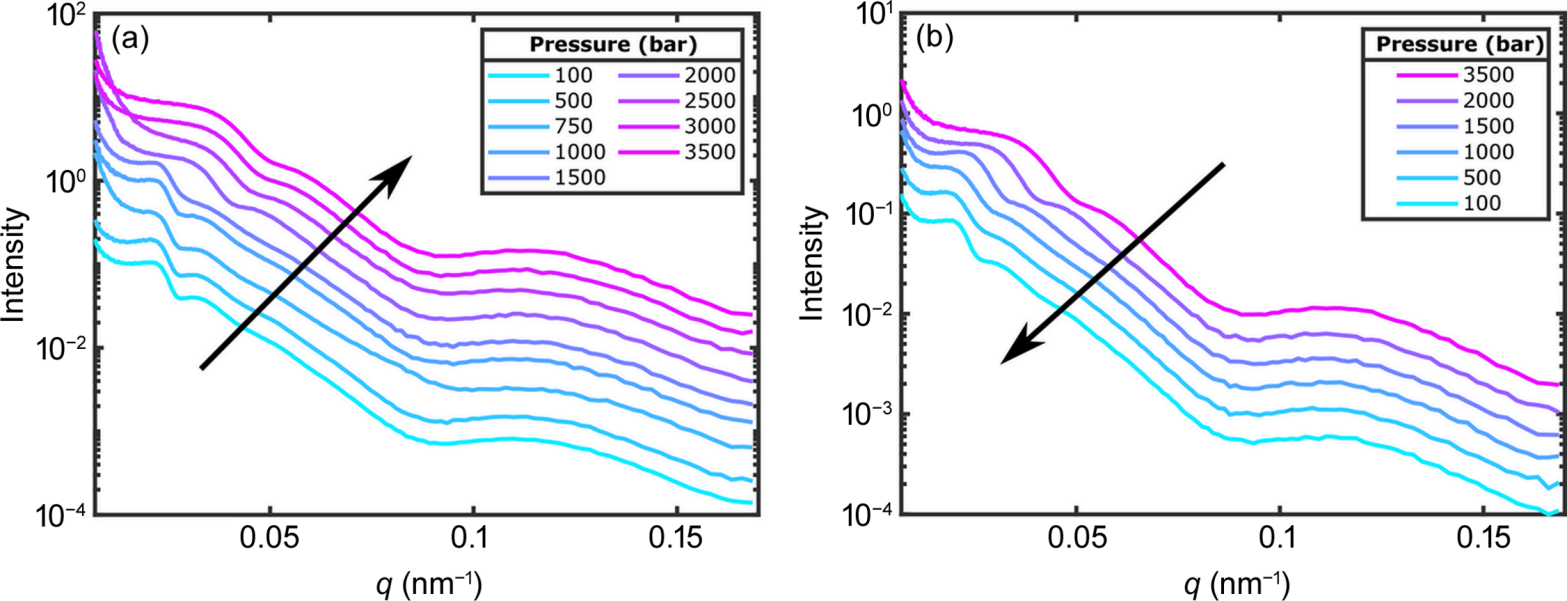
Azimuthally averaged scattered intensity of SP1 for (*a*) increasing pressure and (*b*) decreasing pressure. The intensity profiles have been shifted vertically for clarity.

The azimuthally averaged scattered intensities of the X-ray scattering data are shown for SP1 in Fig. 3 ▸ and for SP2 in the supporting information. The intensity profiles have been shifted vertically for clarity. For *q* ≳ 0.07 nm^−1^ the form factor of the silica core dominates *I*(*q*). As this does not vary with pressure, any effect of pressure on the size or shape of the silica core can be neglected. At low *q*, a change of the first peak at around *q* = 0.025 nm^−1^ is observed between 1500 and 2000 bar upon increasing pressure. When the pressure is decreased, the sample does recover, as shown in Fig. 3 ▸(*b*).

This indicates changes in the structure factor *S*(*q*). Interestingly, the *I*(*q*) profiles resemble the results of temperature-induced volume phase transitions reported by Frenzel *et al.* (2021 ▸). Comparison with those results suggests that the system undergoes a transition from a repulsive, overpacked glass state, found below the LCST and at low pressures, to an attractive, colloidal gel at high temperatures and pressures. However, such structural information is not sufficient to characterize the sample state precisely. Therefore, information about the sample dynamics needs to be obtained by means of XPCS.

**Figure 4:**
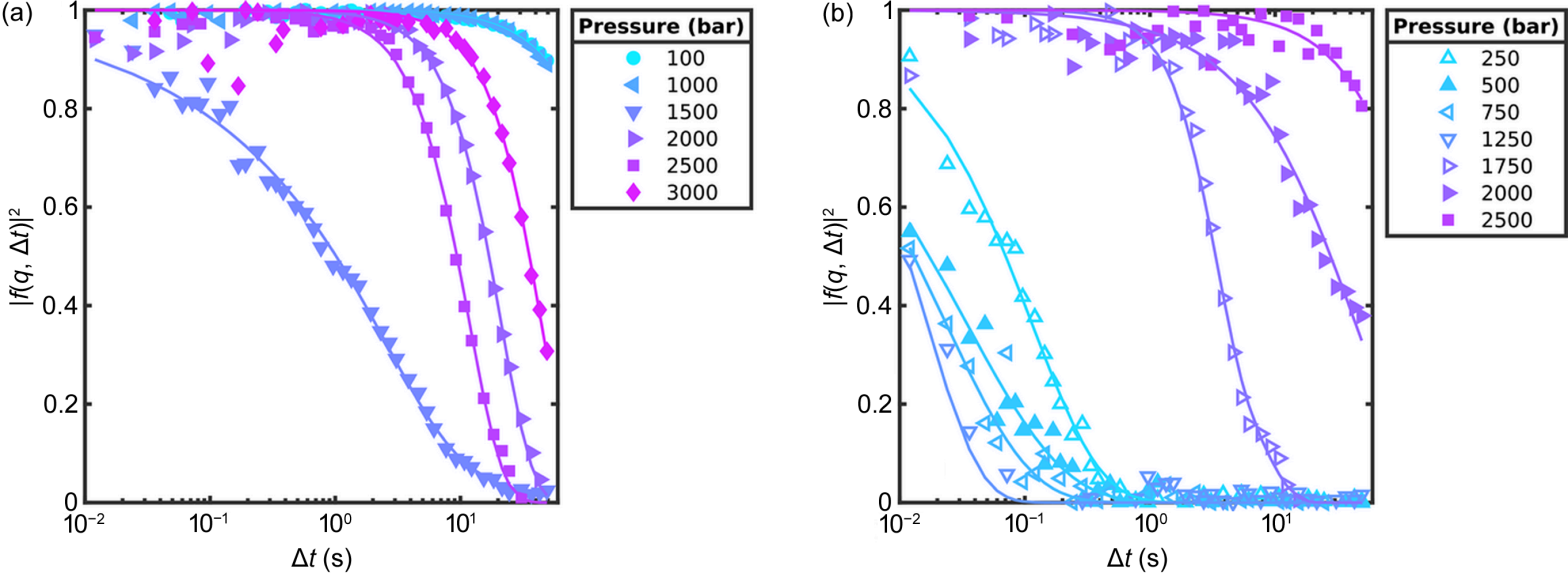
Intermediate scattering functions for (*a*) SP1 and (*b*) SP2 at various pressures for *q* = 0.022 nm^−1^. For clarity not all measured pressures are shown. The lines are fits with equation (3). In the case of 1500 bar for SP1 and 1750 bar for SP2 they are fits with equation (4) with a second decay showing τ > 10 s. Since the statistics are low for Δ*t* < 1 s and the fit only considers values of Δ*t* > 1 s for pressures *p* > 2000 bar of SP2, some values of |*f*(*q*, *t*)|^2^ for Δ*t* < 1 s are not shown due to a high fluctuation.

For the XPCS analysis, the intermediate scattering functions were determined from the *g*_2_-functions and are shown in Figs. 4 ▸(*a*) and 4 ▸(*b*) for SP1 and SP2 at *q* = 0.022 nm^−1^, respectively. The dynamics of SP1 are very slow for pressures up to 1000 bar, then accelerate significantly for *p* ≃ 1500 bar and slow down again for higher pressures. SP2 shows faster dynamics at low pressures, which accelerate with increasing pressures up to 1250 bar. When the pressure is increased further, the dynamics rapidly slow down to relaxation times which are outside our experimental window.

**Figure 5:**
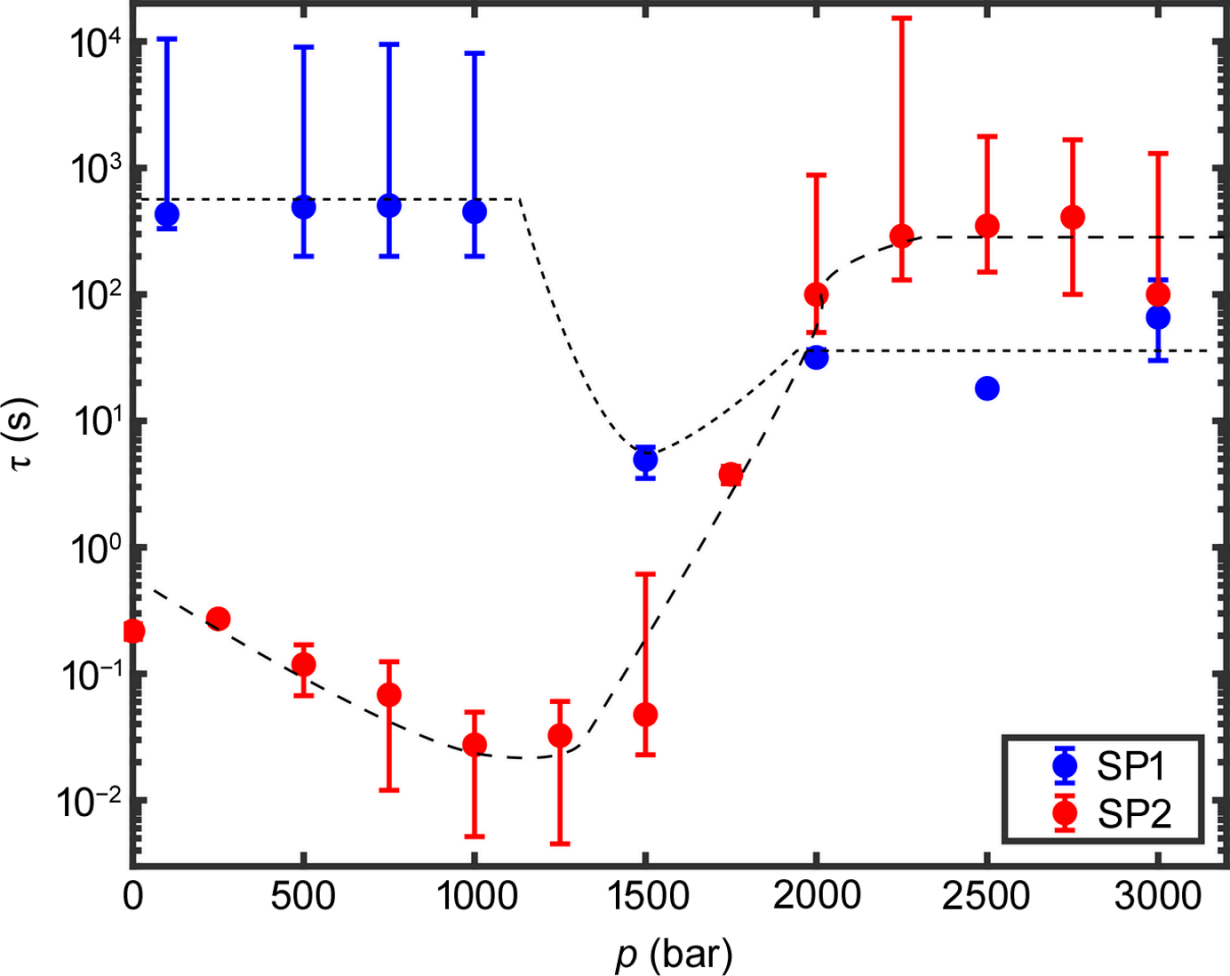
Relaxation times τ for both samples. SP1 is shown in blue, SP2 in red. The dashed lines are guides to the eye.

To further analyze the dynamics of the samples, equation (3) was fitted to the intermediate scattering functions and the resulting relaxation times τ are shown in Fig. 5 ▸ for both samples. At 1500 bar for SP1 and 1750 bar for SP2, the intermediate scattering function showed a double decay and a function of type 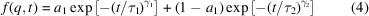
was fitted to the data. These fits are presented in the supporting information. In the following, only the obtained values for the main decay, which in this case is the decay with the smaller relaxation time τ, are shown. For SP1 the relaxation times are larger than 100 s for pressures up to 1000 bar. Since our XPCS measurements only cover a range from 0.012 to 50 s, the *g*_2_-function could not be captured fully and the error on the relaxation times is high in this case. When the pressure is increased even further the relaxation time decreases. In contrast, SP2 showed a different behavior. Here, for low pressures the dynamics were much faster than for SP1 and decreased even further with increasing pressure up to 1000 bar. For pressures *p* > 1500 bar the relaxation time increased rapidly up to more than 100 s and thus was outside our experimental window. The KWW exponents are shown in the supporting information. They are found to be between 1 and 2 for SP1 and between 0.5 and 1.3 for SP2. Both samples show an exception where the sample undergoes the rapid change in dynamics, at 1500 and 1750 bar for SP1 and SP2, respectively.

These results are well in line with our previous temperature-dependent studies. At low pressures sample SP1 shows relaxation times in the range of 1000 s. In combination with the KWW exponent γ > 1 (see the supporting information) this represents a repulsive, overpacked colloidal glass. Upon increasing the pressure, SP1 shows a sudden speed-up to relaxation times in the second range and then slows down at higher pressures. At the same time, *I*(*q*) changes. Both observations, together with the decrease of the KWW exponent to γ ≤ 1 at around 1500 bar and the following increase, suggest a fluidization of the systems expressed by diffusive dynamics followed by dynamics typical for colloidal gels at higher pressures. This is similar to the results discussed by Frenzel *et al.* (2021 ▸) for temperature-induced volume phase transitions. Considering the change of effective volume fraction from above 1 to below 0.05 above the LCST, this suggests that SP1 shows a glass–gel transition at around 1500 bar. For SP2, the dynamics are characterized by stretched correlation functions and speed-up at low pressures. This is indicative of a dense colloidal liquid. This and the sudden slow-down agrees with the results reported by Frenzel *et al.* (2019 ▸). There, a system with an effective volume fraction of 0.55 at 293 K was investigated, which matches the concentration studied in this work. This speed-up of dynamics below the LCST was only found for this concentration range of a dense liquid. At higher concentrations, a glass state is reached, while at low concentrations corresponding to a fluid phase the changes in dynamics reflect the change of temperature and thus the viscosity of the solvent (Frenzel *et al.*, 2021 ▸). Furthermore, both systems show a double decay in the transition regime, *i.e.* at 1750 bar in this study and 310 K in that by Frenzel *et al.* (2019 ▸). This indicates a pressure-induced transition from a dense liquid phase to a colloidal gel at around 1750 bar. Remarkably, for both samples the phase transition of the colloidal system takes place at lower pressures than the volume phase transition in the single-particle limit, which was reported to be around 2000 bar (Niebuur *et al.*, 2020 ▸; Papadakis *et al.*, 2023 ▸). This may be a consequence of the high particle concentration and thus a potential incomplete hydration of the PNIPAm shells, especially reported at high concentrations (Scotti *et al.*, 2019 ▸; Scotti *et al.*, 2022 ▸). Notably, the KWW exponent is smaller for SP2 compared with SP1, while its relaxation time shows slower dynamics. The small γ suggest a higher degree of dynamical heterogeneity in this system, potentially reflecting a different gel state at the lower volume fraction compared with SP1. Note that slower dynamics for a gel formed at lower volume fractions has also been reported for the temperature-induced studies (Frenzel *et al.*, 2021 ▸).

During the experiment, we measured XPCS runs in batches in order to gain more statistics. A typical measurement consists of 10 batches where each batch consists of a series of 6000 speckle patterns. The first batch was measured after the target pressure has been reached. The results shown up to this point were all obtained by analyzing only the last batch. Even though the change in pressure in the experimental setup is instantaneous, the dynamics in the sample take some time to adjust. This equilibration process can be investigated by analyzing each batch separately and is presented in the following.

**Figure 6:**
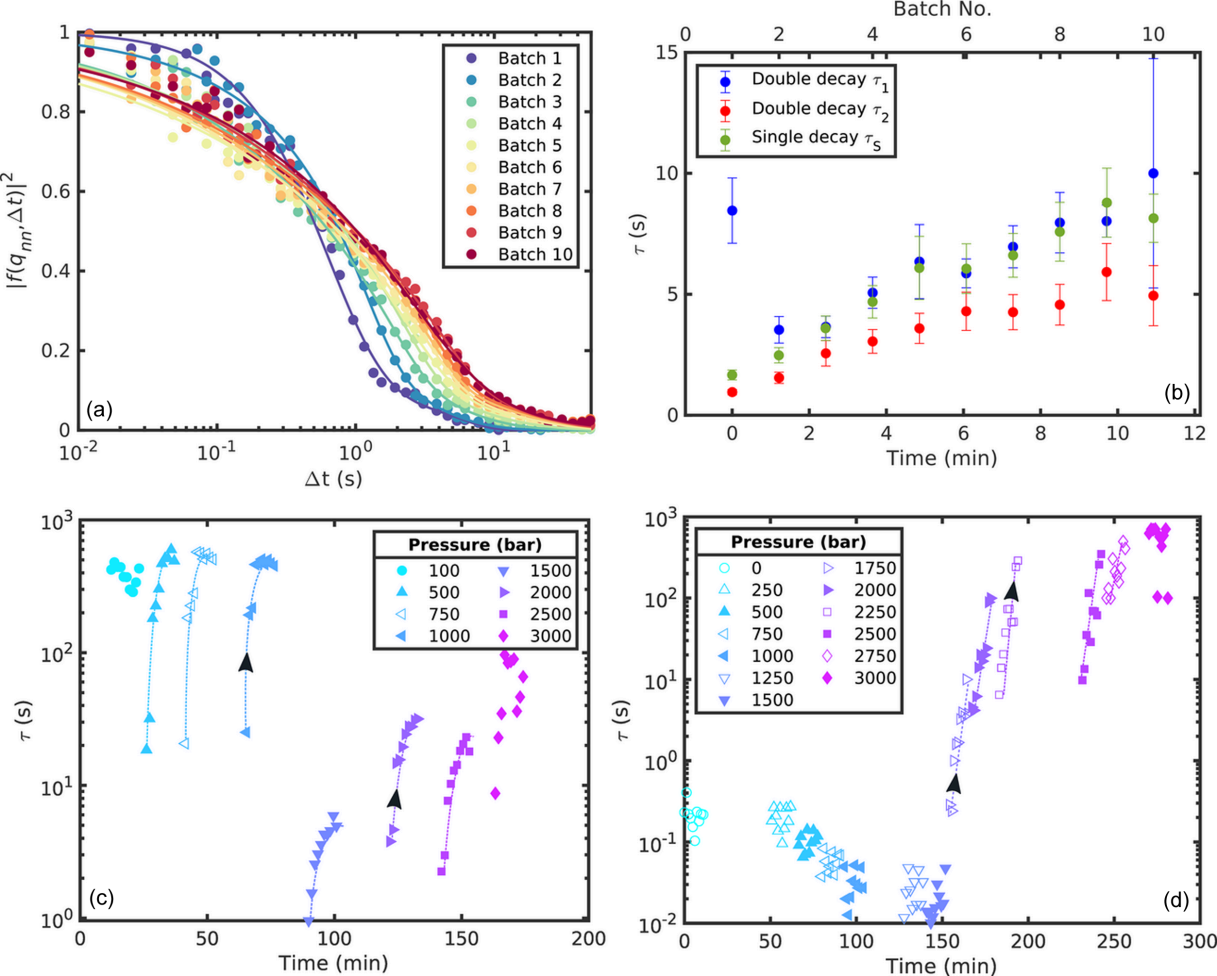
Batch-dependent analysis. (*a*) Intermediate scattering functions and (*b*) relaxation times for different batch numbers of SP1 at 1500 bar. (*c*) and (*d*) Relaxation time τ as function of the total experimental time for SP1 and SP2, respectively. The dashed lines are guides to the eye; the black arrows show the direction with increasing pressure.

The intermediate scattering functions for the different batches are shown in Fig. 6 ▸(*a*) for SP1 at 1500 bar. There is a clear change in relaxation time as well as shape of the intermediate scattering function with batch number, indicating that it takes some time for the sample to adjust to the increased pressure. Since the intermediate scattering functions for this pressure showed a double decay, equation (4) was fitted to these data. Additionally, the main decay was fitted with equation (3). The obtained relaxation times are shown for each batch in Fig. 6 ▸(*b*). They all follow the same trend; τ increases with increasing waiting time and reaches a plateau for the last batches, suggesting that the sample has fully adjusted to the pressure for the last batches. The relaxation time values from the single decay fit are between the values from the double decay. The KWW exponents γ, which are shown in the supporting information, decrease from around 1 for the first batch to 0.5 for the last batches as the intermediate scattering function becomes more and more stretched. The second KWW exponent from the double decay remains γ_2_ > 1 for all batches.

Additionally, Figs. 6 ▸(*c*) and 6 ▸(*d*) show the relaxation time τ of the individual batches as a function of the total experimental time for all measured pressures. For SP1 at 100 bar, the relaxation time of the different batches does not vary much since the system had been at that pressure for long enough to equilibrate. However, upon increasing the pressure up to 1000 bar, a strong batch dependence of the relaxation time can be observed. With each increase in pressure, the relaxation time decreases to values of below 30 s for the first batch, increasing again over the next few batches over the duration of a few minutes to reach a value of τ ≃ 500 s for the last batches. When the pressure was increased to 1500 bar, the relaxation time decreases significantly to τ < 10 s but shows the same batch-dependent behavior as before. For SP2, we observe no batch dependence for pressures up to *p* =1500 bar. For pressures *p* > 1500 bar the same batch-dependent behavior as for SP1 can be found. For *p* = 1750–2500 bar, τ increases by more than one order of magnitude with increasing batch number. This suggests that the change of dynamics upon pressure change only appears when the system is in a glass or gel state. As SP1 shows a transition from a colloidal glass towards a gel for higher pressures, this equilibration process as a response to the induced pressure is present for all pressures. In contrast, SP2 is in a liquid state for *p* < 1750 bar, where no batch dependence is observed. Once a gel state is reached, τ is again batch dependent.

The kinetics of the pressure-induced coil-to-globule transition in PNIPAm have been studied by SANS by Niebuur *et al.* (2018 ▸, 2019*a* ▸), reporting on structural changes of a polymer solution taking more than 10^2^ s before the final state is reached. For micro- and nanogels, the timescale of temperature-induced transition has been reported to be in the sub-millisecond range (Zhao *et al.*, 2018 ▸; Dallari *et al.*, 2024 ▸). In contrast to these studies, we focus here on the pressure response of the whole colloidal system. While the dynamics show aging (*i.e.* slow down after a pressure change), the structure expressed by *I*(*q*) does not vary (see the data shown in the supporting information). Such aging of the dynamics has been found in many glass and gel formers. Its absence in the liquid state and the occurrence of a two-step decay with a KWW exponent of around 2 suggests that this aging is driven by the release of stress upon pressure change (Dallari *et al.*, 2020 ▸). After about 10 min the stress is mostly released and the dynamics represent the typical behavior of a colloidal glass or gel, respectively.

## 4. Conclusions

We determined the pressure-dependent structure and dynamics of concentrated silica-PNIPAm nanogels. The XPCS results show a similar behavior to previous temperature-dependent studies. Upon increasing the pressure, the 6.5% mass fraction sample SP1 shows a transition from a colloidal glass with slow dynamics to a colloidal gel. SP2, twice as dilute as SP1, is in a liquid state at low pressures and shows a transition to a gel state for *p* > 1750 bar. During the transition both systems show a double decay, which is also observed for temperature-induced transitions (Frenzel *et al.*, 2019 ▸). However, while we observe a broad transition pressure range of a few 100 bar, for temperature the range is only about 1 K (Frenzel *et al.*, 2019 ▸; Frenzel *et al.*, 2020 ▸; Frenzel *et al.*, 2021 ▸). Despite the different driving forces of temperature- and pressure-induced transitions of PNIPAm, the phase behavior of the colloidal system shows similarities with respect to structure and dynamics. This indicates that the underlying process does not influence the phase of the colloidal system which forms fluid, glassy and gel phases depending on the concentration and pressure. In this way our study is a first step to expand the phase diagram for this important system. Additionally, we investigated the response of the sample to a pressure change by performing a time-dependent analysis of our XPCS data. The response is immediate for the fluid states and takes a few minutes for glasses and gels, even when raising the pressure step-wise. We also observed that, for a system in the glass and gel state, the relaxation time always decreases first on increasing the pressure. This aging seems to be driven by stress release and motivates follow-up studies both experimentally and theoretically, since our understanding is still limited (*e.g.* Tavagnacco *et al.*, 2021 ▸). On the single-particle level the temperature-induced volume phase transition of PNIPAm was found to take place on a (sub-)microsecond timescale (Dallari *et al.*, 2024 ▸). To the best of our knowledge, the kinetics of pressure-induced transitions have only been investigated for PNIPAm solutions (Niebuur *et al.*, 2019*a* ▸; Niebuur *et al.*, 2018 ▸); data on micro- or nanogels are missing. In this context, our present work contributes to close this gap for colloidal systems, focusing on interparticle interactions.

## Supplementary Material

Scattered intensities, double decay fits and KWW exponents. DOI: 10.1107/S1600576725003188/jl5104sup1.pdf
