# Supplementary material for: Phase behavior of silica-PNIPAm nanogels under high hydrostatic pressure
Source: J Appl Crystallogr. 2025 May 12;58(Pt 3):919–26. doi: 10.1107/S1600576725003188 (PMC12135980; doi:10.1107/S1600576725003188)
Supplement: Supplementary file 1 [file j-58-00919-sup1.pdf]

Supplementary Information: Phase behavior of Silica-PNIPAm nanogels under high hydrostatic pressure

Nele N. Striker,[a*] Christina Krywka,[b] Claudia Goy,[a] Svenja C. Hövelmann,[a,c] Niels C. Giesselmann,[a] Florian Schulz,[d] Irina Lokteva,[a,f] Fabian Westermeier,[a] Frederic Caupin,[e] Michael Paulus[g] and Felix Lehmkühler [a,f*]

[a]Deutsches Elektronen-Synchrotron DESY, Notkestr. 85, 22607 Hamburg, Germany, [b]Helmholtz-Zentrum Hereon, Institute for Materials Physics, Max-Planck-Str. 1, 21502 Geesthacht, Germany, [c]Institute of Experimental and Applied Physics, Kiel University, Leibnizstraße 19, 24118 Kiel, Germany, [d]Fachbereich Physik, Universität Hamburg, Luruper Chaussee 149, 22761 Hamburg, Germany, [e]The Hamburg Centre for Ultrafast Imaging, Luruper Chaussee 149, 22761 Hamburg, Germany, [f]Institut Lumière Matière, Université Claude Bernard Lyon 1, CNRS, Institut Universitaire de France, F-69622 Villeurbanne, France, and [g]Fakultät Physik/DELTA, TU Dortmund, 44221 Dortmund, Germany. E-mail: nele.striker@desy.de, felix.lehmkuehler@desy.de

1. Scattered Intensity

The azimuthally averaged scattered intensities for SP2 are shown in Fig. 1. The intensity profiles have been shifted vertically for clarity. They show the same behavior with induced pressure as for SP1. For $q > 0.07$ nm^{-1} the form factor of the silica core dominates and no effect of pressure is observed. At low q, a change in the first

peak at around $q = 0.025$ nm^{-1} is observed between 1500 and 2000 bar upon increasing pressure. When decreasing the pressure, the sample does recover, as is shown in Fig. 1(b).

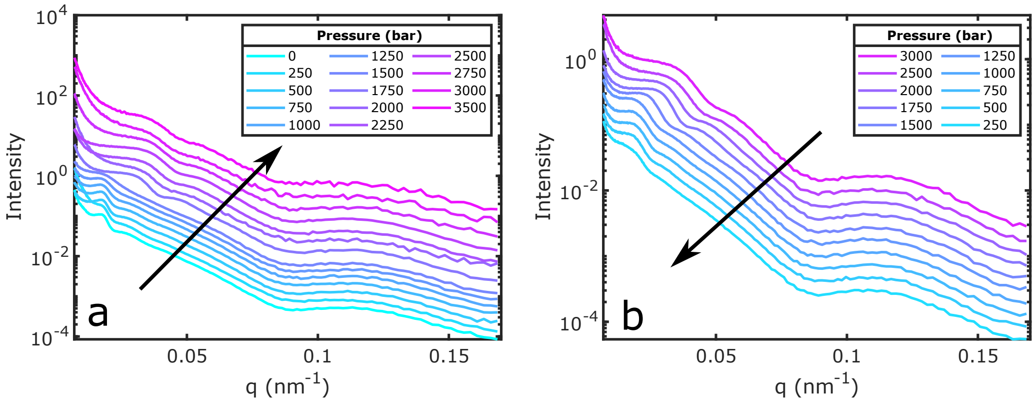

Fig. 1. Azimuthally averaged scattered intensity of SP2 for (a) increasing pressure and (b) decreasing pressure. The intensity profiles have been shifted vertically for clarity.

2. Double decay fits

A double decay is only observed for a single pressure, at 1500 bar for SP1 and 1750 bar for SP2. The double decay fits for the samples at these pressures are shown in Fig. 2 in more detail. Note that $f(q_n n, t)$ and not $|f(q_n n, t)|^2$ is shown. Additionally to the data and fit, the contributions to the fit are shown separately. The coefficients from Eq. 4 are $a_1 = 0.719$ and $a_1 = 0.5368$ for SP1 and SP2, respectively.

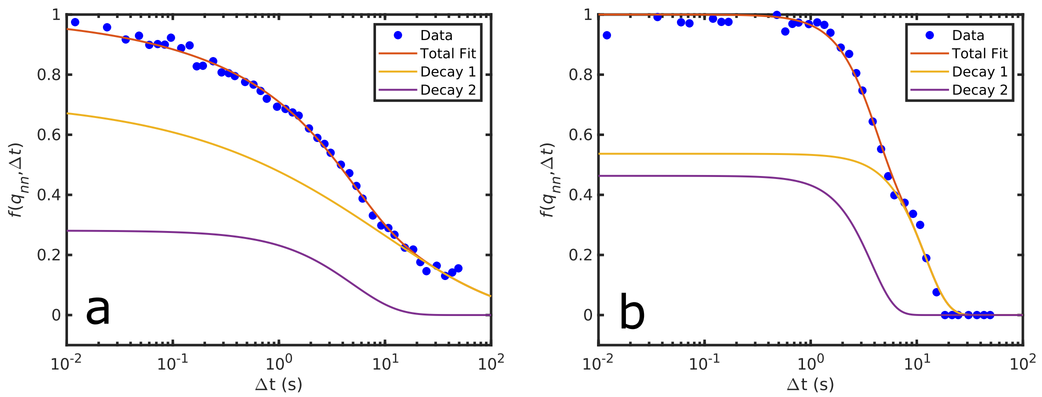

Fig. 2. Double decay fit showing the individual contributions of the two decays for (a) SP1 at 1500 bar and (b) SP2 at 1750 bar.

3. KWW exponents

The KWW exponents for sample SP1 and SP2 are shown in Fig. 3. They were obtained by fitting an exponential of type $\exp(-(x/\tau)^{\gamma})$ to the intermediate scattering functions. For SP1, we find $\gamma > 1$ for low pressures. At $p = 1500$ bar γ decreases to 0.5 and then increases to $\gamma > 1.5$ for higher pressures. For SP2, γ increases for low pressures, then decreases rapidly to 0.5 for $p = 1500$ bar and increases again.

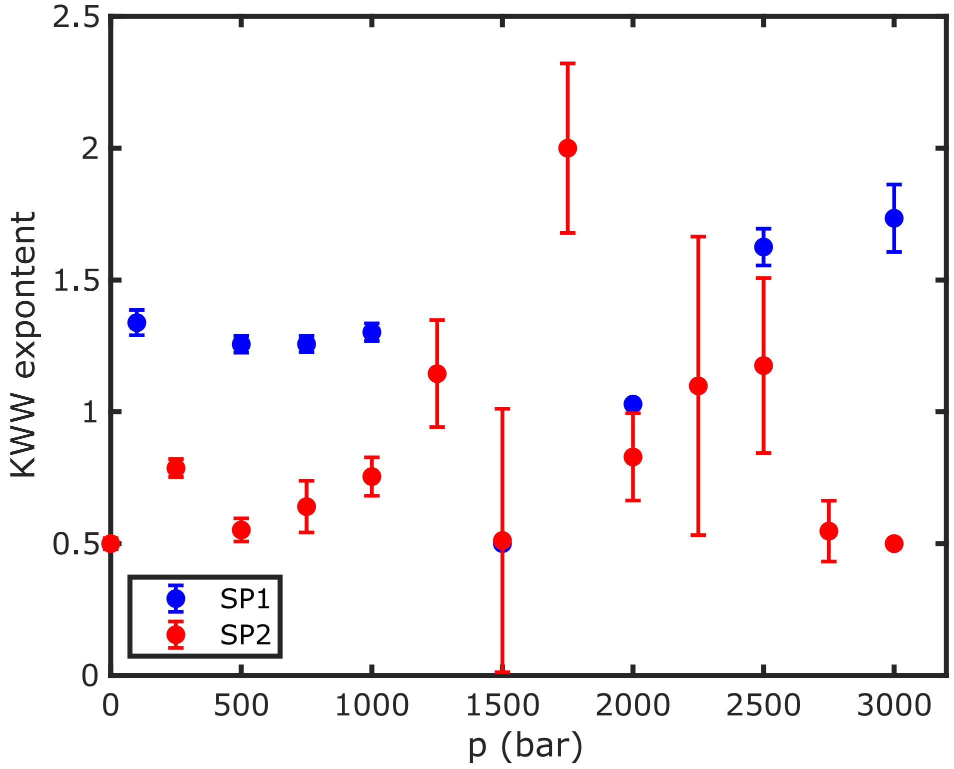

Fig. 3. KWW exponents γ for SP1 and SP2 at $q = 0.022$ nm^{-1}.

4. Batch-dependent analysis: Intensity and KWW exponents

The batch-dependent azimuthally averaged scattered intensity is shown in Fig. 4 for SP1 and SP2 at p = 1500 bar and 1750 bar, respectively. At these pressures the systems undergo a rapid change in dynamics and for SP2, this is the first pressure where aging was observed. No change with increasing waiting time is observed except for the first batch of SP1, where the intensity is higher than for the following batches for $q < 0.01$ nm^{-1}.

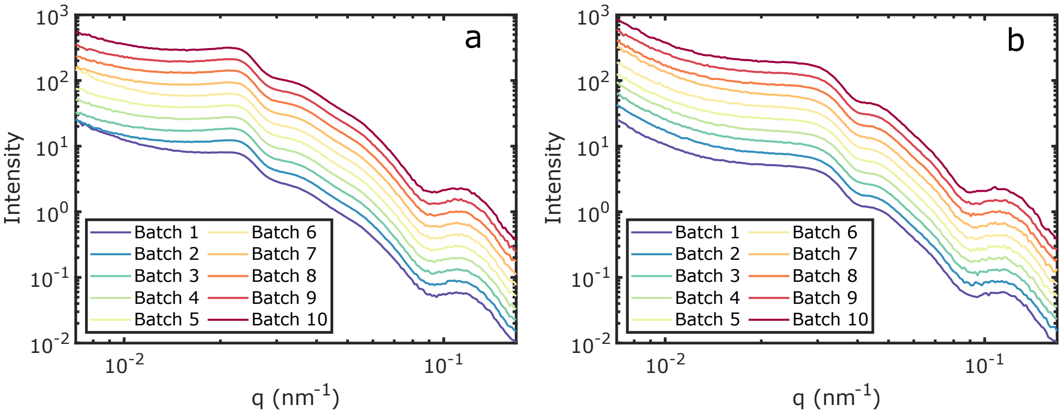

Fig. 4. Batch-dependent azimuthally averaged scattered intensity for (a) SP1 at $p =$ 1500 bar and (b) SP2 at 1750 bar. The intensity profiles have been normalized and are shifted vertically for clarity.

The KWW exponents γ from both the double and the single decay fits for sample SP1 at 1500 bar are shown in Fig. 5. For γ_2 no errorbars are shown since the fit runs into its limit at $\gamma = 2$. The first KWW exponent of the double decay γ_1 and the KWW exponent of the single decay fit γ_S show a very similar behaviour, in the beginning of the measurement series γ decreases to $\gamma \approx 0.5$ and then stays constant for the remaining measurements. The second KWW exponent of the double decay fit is much higher with $\gamma_2 > 1$.

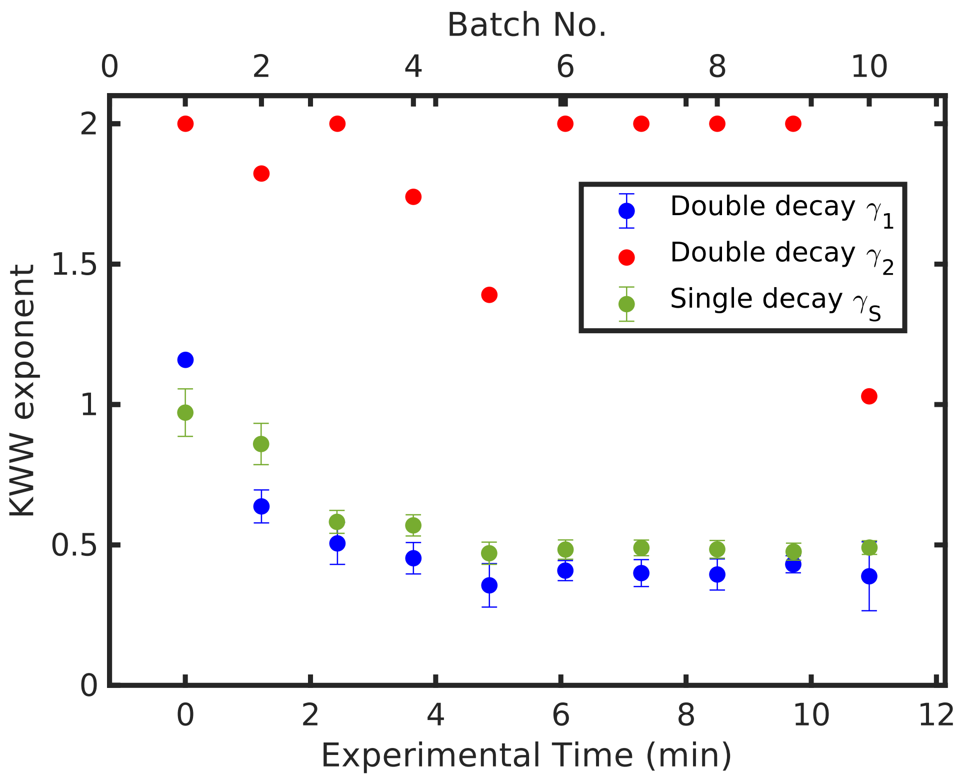

Fig. 5. KWW exponents γ of SP1 at 1500 bar for fits with a double decay and a single decay as a function of the experimental time and the batch number. For γ_2 of the double decay fit no errorbars are shown since the fit runs into its limit at $\gamma = 2$.
